# 128-Slice Acceletated-Pitch Dual Energy CT Angiography of the Head and Neck: Comparison of Different Low Contrast Medium Volumes

**DOI:** 10.1371/journal.pone.0080939

**Published:** 2013-11-19

**Authors:** Yu Chen, Huadan Xue, Zheng-yu Jin, Jie Zhang, Hao Sun, Xuan Wang, Zhu-hua Zhang, Da-ming Zhang, Guang-ming Lu, Zhao-qi Zhang, U. Joseph Schoepf, Andreas M. Bucher, Christopher D. Wolla, Yun Wang

**Affiliations:** 1 Department of Radiology, Peking Union Medical College Hospital, Chinese Academy of Medical Sciences, Beijing, China; 2 Department of Medical Imaging, Jinling Hospital, Clinical School of Medical College, Nanjing University, Nanjing, Jiangsu, China; 3 Department of Radiology, Beijing Institute of Heart, Lung and Blood Vessel Diseases and Beijing Anzhen Hospital, Capital Medical University, Beijing, China; 4 Department of Radiology and Radiological Science, Medical University of South Carolina, Charleston, South Carolina, United States of America; Institute of Automation, Chinese Academy of Sciences, China

## Abstract

**Background:**

Our study aims to evaluate the image quality and feasibility of 128-slice dual-energy CTA (DE-CTA) for supra-aortic arteries using reduced amounts of contrast medium (CM).

**Methods:**

A prospective study was performed in 54 patients receiving CTA of the head and neck with a 128-slice dual-source CT system. Patients were randomized into two groups with a volume of either 40 mL of CM (Group I) or 50 mL of CM (Group II). Arterial and venous enhancements were recorded for quantitative assessment. Qualitative assessments for images without bone removal (BR) were based on a) the visualization of the circle of Willis and b) streak artifacts due to residual CM in the subclavian or internal jugular veins ipsilateral to injection of CM. Qualitative assessment of dual-energy images using BR was based on the presence of bone remnants and vessel integrity. Quantitative data was compared using the Student *t* test. The χ^2^ test was used for the qualitative measurements of streak artifacts in veins while the Mann-Whitney U test was used for the qualitative measurements of images with BR.

**Results:**

Arterial and venous attenuation was significantly higher in Group II (*P*=0.000). Image quality regarding the circle of Willis was excellent in both groups (3.90±0.30 for Group I and 4.00±0 for Group II) . Imaging of the internal jugular veins was scored higher in Group I (1.87±0.72) compared with Group II (1.48±0.51) (*P*=0.021). Within Group I using BR, mean scores for bone remnants did not differ significantly (*P*>0.05) but mean scores of vessel integrity (*P*<0.05) did.

**Conclusions:**

Contrast-enhanced head and neck CTA is feasible using a scan protocol with low amounts of contrast medium (40 mL) on a 128-slice dual-energy CTA. The 40-mL protocol provides satisfactory image quality before and after dual-energy bone-removal post-processing.

## Introduction

Computed tomography angiography (CTA) is a fast and often readily available imaging technique of cerebral and carotid arteries, next to Magnetic Resonance Angiography [1,2]. However in CTA the depiction of bone and calcifications can impair assess-ability of the images [3]. Dual energy CTA (DE-CTA) however, can separate iodine from bone by their different absorption spectrums [4,5]. Because it has a two-X-Ray-tube system, DE-CTA is originally associated with a higher radiation-dose than single-energy CTA[6,7]. More recent improvements in DE scanning techniques have been realized with the second-generation dual-source CT (DSCT) system [8]. Because of the wider detector and higher pitch, DE-CTA performed with the second generation DSCT could actually result in lower radiation dose and faster scanning speed[8,9], which in turn would require less volume of contrast medium.

One group of Patients that would especially benefit from a reduced amount of CM are those with Chronic Kidney Disease (CKD) [10,11]. At the same time patients with renal insufficiency exhibit a significantly higher percentage of calcifications compared with patients that do not exhibit chronic kidney disease (CKD) (21.2±16.1 % vs. 10.9±9.8 %) [12,13]. 

Another expected benefit would be that artifacts occurring due to CM in the subclavian and jugular veins (SV, JV) ipsilateral to injection, which can impair image quality, could be reduced by a shorter bolus injection[14] and venal flushing with saline solution[15].

To our best knowledge there has been no reports using dual-energy protocols with administration of less than 50-mL CM for CTA of the head and neck. This is the first evaluation of both the vascular enhancement and image quality of dual-energy bone removal using a 128-slice dual energy CT scan. The aim of this study is to evaluate image quality as well as feasibility of head and neck dual-energy CTA with a reduced amount of CM at an acceletated-pitch 128-slice Dual Source CT. 

## Materials and Methods

### Study Population

Ethical approval was given by the local ethics committee of Peking Union Medical College Hospital. This prospective study was performed from July to November in 2011 and all patients signed informed consent documents for the entire procedure. 59 patients with suspected atherosclerotic disease of the carotid or vertebra-basilar arteries were referred to head and neck CTA in our institution during the study period. Exclusion criteria included: known allergic reaction to iodinated contrast medium (n=2), impaired renal function (serum creatinine level ≥ 132 μmol/L, (n=2), severe cardiac insufficiency (n=1), pregnancy (n=0), and age under 18 years (n=0). At the conclusion of the study, 54 consecutive patients were enrolled, and demographic characteristics (sex, age, height and weight) were recorded for each enrolled patient.

### Scanning Protocol

According to the randomization formula [16], all patients were assigned to one of the two injection protocols (Group I: 40 mL CM, Group II: 50 mL CM).

CTA was performed with a 128-slice dual source CT system (Somatom Definition FLASH, Siemens Healthcare, Forchheim, Germany). Contrast medium (Ultravist, 370 mg of iodine per mL, Bayer-Schering HealthCare, Germany) was injected with a dual-head power injector (Empower, EZEM Inc., New York, U.S.A.). The contrast medium was injected through an 18-gauge intravenous (IV) antecubital catheter at a rate of 6 mL/s followed by a bolus chaser of 48-mL saline. The enhanced scan was performed in a caudal to cranial direction with the scan range reaching from the aortic arch to the apex of the skull. Study acquisition delay time was estimated by initial injection of a 5-mL CM test bolus at 6 mL/s followed by 36-mL saline. The low-dose (80 kV, 45 mAs) dynamic monitoring scans were positioned at the level of the carotid bifurcation. Acquisition of the dynamic monitoring scans started 10 s after the beginning of the injection. The delay time between each monitoring scan acquisition was 0.5 s and the cycle time was 1 s. 

A region-of-interest (ROI) tracer was placed within the common carotid artery (CCA). Scanning was stopped when attenuation in this ROI decreased. Images of the test bolus were sent to the ‘Dynamic Evaluation’ software card on the workstation (Somaris/7 syngo CT 2011A, VA40A, Siemens Healthcare, Forchheim, Germany). Then a second ROI was placed inside the bifurcation of the common carotid artery to measure the corresponding peak of maximum enhancement (PME). The delay time was calculated as time to PME (tPME) plus 2 s for the 40-mL group and 3 s for the 50-mL group. The time interval between test-bolus and contrast enhancement scan was 20-30 s. 

We performed DE-CTA with the following parameters: CareDose-Mode: on, 104 reference mAs at both 100 kVp (Tube A) and Sn140 kVp (Tube B), 0.28 s rotation time, 64×2×0.6 mm collimation with a z-flying focal spot, and a pitch of 1.2. All images were reconstructed with slice thickness of 1.0 mm, slice interval of 0.9 mm, 512×512 matrix and individually adapted field of view (FOV = 236-286 mm). Three image data sets including 100 kVp, Sn140 kVp, and dual-energy weighted data set (DE composition=0.6) roughly equivalent to a conventional 120 kV scan were reconstructed from each dual energy (DE) scan. CT dose index volume (CTDI_vol_) and dose-length product (DLP) were recorded from the scan protocol of each patient. 

### Image post-processing

100 kV and Sn140 kV data sets were transferred into a workstation (Syngo MMWP, VE32D, Siemens Healthcare, Forchheim, Germany). Dual energy based bone removal was performed using the “head bone removal” presets (low kV and high kV soft tissue attenuation, 50 HU; ratio 1.90; minimum 130 HU, maximum 700 HU; filter range, two pixels; blooming reduction and fragment removal activated) in the ‘Dual Energy’ software card. 

### Image assessment

A 2-year attending and 3-year associated professor with experience in vascular imaging performed the qualitative analysis. Both readers were blinded to the CT protocol used for the images. Presence of steno-occlusive disease in arteries was calculated according to the North American Symptomatic Carotid Endarterectomy Trial criteria and categorized in mild (<50%), moderate (50%–69%), severe stenosis (70%–99%), and complete occlusion (100%) [17,18] according to the axial 1.0-mm enhanced multi-planar reformation (MPR) images from the dual-energy weighted data sets. 

#### Quantitative assessment

MPR images from the dual-energy weighted data sets were used for attenuation measurements in ‘3D’ software card on the workstation (Syngo MMWP, VE32D, Siemens Healthcare, Forchheim, Germany). The locations of the measurements were recorded as follows: aortic arch (AA), proximal subclavian arteries (SA), bifurcation of common carotid arteries (CCA), siphon of internal carotid arteries (ICA), proximal segment (M1) of middle cerebral arteries (MCA), basilar artery (BA), superior vena cava (SVC), straight sinus (SS), and internal jugular vein (IJV_contra_) on the contralateral side of the CM injection. SVC, IJV_contra_ and SS were measured at the same level of the AA, CCA and BA, respectively. To avoid partial volume effects, both reviewers placed the ROIs in individually adapted MPR images precisely perpendicular to the vessel lumen. Whenever possible, ROI measurements were performed on 3 continuous slices in central parts of the vessel without pathologic findings. In cases of severe stenosis or heavy calcification, the vessel cavity attenuation was measured distal to the stenotic or calcified part of the vessel. Completely occluded or aplastic segments were not evaluated. Mean value of the three measurements on the same vessel was calculated.

#### Qualitative assessment

Both images of standard reconstruction without bone removal (BR) and dual-energy reconstruction with BR underwent qualitative assessment. For images from dual-energy weighted data sets without BR, evaluations of intracranial arteries (Circle of Willis) and streak artifacts due to residual CM in veins (SV and IJV) ipsilateral to side of injection were performed. 

On the evaluation of intracranial arteries, visual assessment of the Circle of Willis was achieved using branches of the MCA. The maximum intensity projections (MIP) of MCA branches were selected for evaluation of vessel visualization using a 4-point scale as follows [19]:

- S_willis_ = 1 poor; incomplete demonstration of M1
- S_willis_ = 2 insufficient; complete demonstration of M1 but no demonstration of M2 or distal branches
- S_willis_= 3 sufficient; incomplete demonstration of M2 and distal branches
- S_willis_= 4 excellent; complete demonstration of M3-4 branches.

Streak artifacts due to concentrated CM in the subclavian veins (SV_ipsi_) and internal jugular veins (IJV_ipsi_) ipsilateral to side of injection were rated using a 3-point scale as follows [14]:

- S_vein_ = 1 artifact would impair evaluation of adjacent vessels
- S_vein_ = 2 artifact does not impair the evaluation of adjacent vessels
- S_vein_ = 3 no artifact

For images of dual-energy reconstruction with BR, the two readers separately performed qualitative assessments including the bone remnants and vessel integrity. The thin slab MIP (slab thickness 30mm) technique was selected for the assessments. Bone removal was rated used a 3-point scale adapted from Lell’s study [16] as follows:

- S_bone_ = 1 poor; bone remnants longer than 1 cm in the longest axis
- S_bone_= 2 sufficient; only small bone remnants less than 1 cm
- S_bone_= 3 excellent; no or irrelevant bone remnants

And the following score was used for vessel integrity [20]:

- S_integrity_ = 1 poor; gaps within the vessel
- S_integrity_= 2 insufficient; artificial lumen reduction of the vessel resulting in hemodynamically significant stenosis (≥50%)
- S_integrity_= 3 sufficient; artificial lumen alteration resulting in total lumen reduction <50%
- S_integrity_= 4 good; lumen alterations <10%
- S_integrity_= 5 excellent; no lumen alteration.

**Figure 1:**
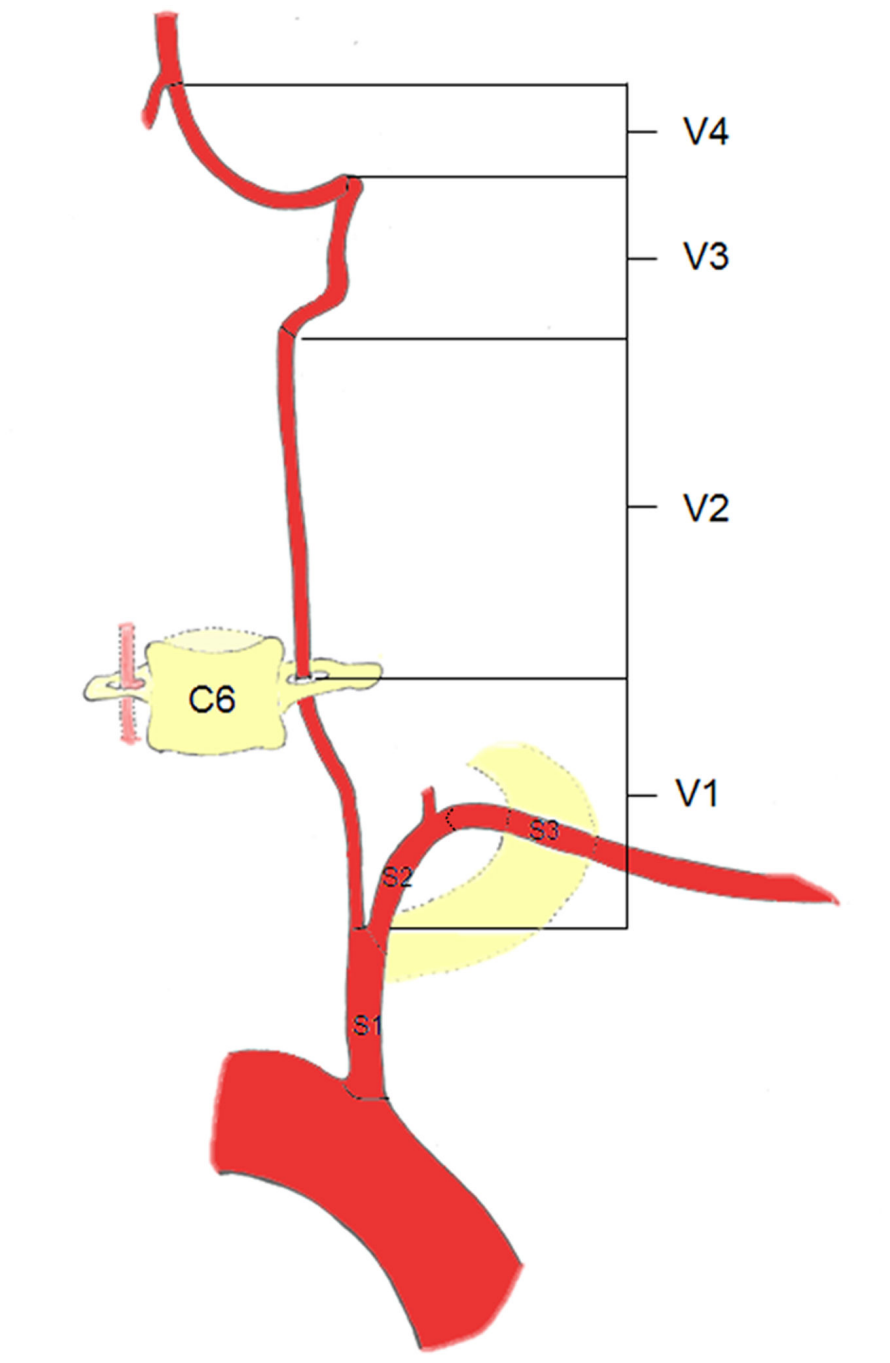
Schematic diagram illustrating the segments of the vertebral artery (VA) and subclavian artery (SA). V1 begins from the origin of VA to where it enters the transverse foramina at the fifth or sixth cervical vertebra. V2 begins from the level of the fifth or sixth cervical to the second cervical vertebra travelling through the transverse foramina at each vertebral level. V3 extends between the C2 transverse process and ends at the base of the skull where it enters the foramen magnum. V4 extends from the point at which the arteries enter the dura to the termination of both VAs at the vertebrobasilar junction. S1 begins the origin of SA and ends just proximal to the origin of SA. S2 begins just proximal to the origin of SA and ends the medial border of the scalenus anterior. S3 extends from the medial border of scalenus anterior to the outer border of the first rib.

**Figure 2:**
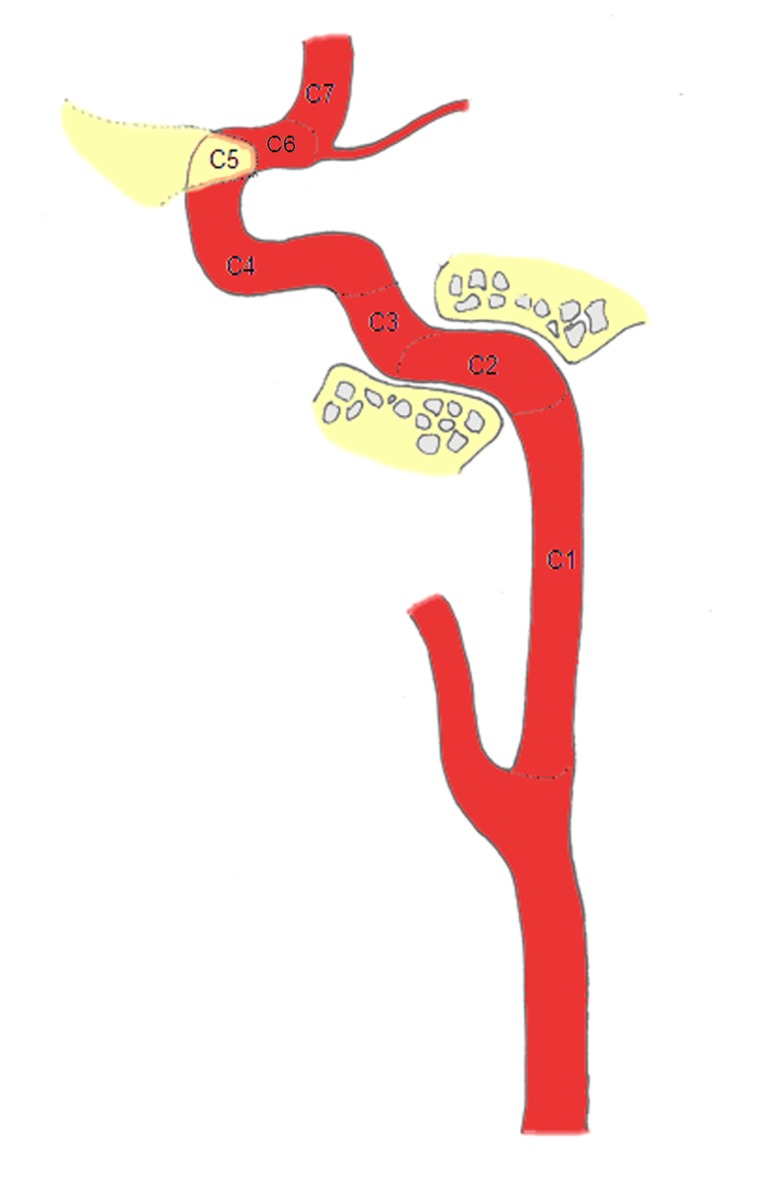
Schematic diagram illustrating the segments of the internal carotid artery (ICA). C1 (cervical segment) begins at the level of the common carotid artery bifurcation to the entrance level of the carotid canal. C2 (petrous segment) extends within the carotid canal. C3 (lacerum segment) begins where the carotid canal ends and ends at the superior margin of the petrolingual ligament. C4 (cavernous segment) is surrounded by the cavernous sinus. C5 (clinoid segment) begins at the proximal dural ring and ends at the distal dural ring where the ICA extends through the medial side of anterior clinoid process. C6 (ophthalmic segment) begins at the distal dural ring and ends just proximal to the origin of the posterior communicating artery (PComA). C7 (communicating segment) begins just proximal to the origin of the PComA and ends at the ICA bifurcation.

In order to provide uniform reference for the two readers, a schematic drawing of the vascular tree with markings of the different vessel segments was provided to assign quality scores to each individual segment (n = 43 segments; aortic arch, brachiocephalic trunk, bilateral S1-3 segments of SA, bilateral external carotid arteries (ECA) and branches, bilateral CCA, bilateral bifurcation of CCA, bilateral C1-7 segments of the ICA [21], bilateral anterior cerebral arteries (ACA), bilateral MCA, bilateral posterior cerebral arteries (PCA), bilateral V1-4 segments of the vertebral arteries (VA) [22] and basilar artery (BA)). (Figure 1 and 2)

### Statistical analysis

The student *t* test was used for comparison of age, height, weight, scan time, CTDI_vol_, DLP, and the vascular enhancement in the two groups after normal distribution was proved by a Kolmogorov-Smirnov test. The χ^2^ test was used to compare sex, location of injection (left or right), arterial stenosis and the qualitative scores of streak artifacts in the SV_ipsi_ and IJV_ipsi_. Qualitative scores of the bone remnants and vessel integrity with BR averaged by both readers were compared with a nonparametric rank sum test (Mann-Whitney U). 

Inter-observer variability for qualitative scores of bone remnants and vessel integrity after BR processing was determined by kappa statistics with the scale as follows:

- Poor agreement, κ=0
- Slight agreement, κ=0.1-0.2
- Fair agreement, κ=0.21-0.4
- Moderate agreement, κ=0.41-0.6
- Good agreement, κ= 0.61-0.8
- Excellent agreement, κ=0.81-1.

Statistical analysis was performed using SPSS Version 17.0 (SPSS Inc., Chicago, IL). A P value of less than 0.05 was assumed to indicate statistical significance. 

## Results

### Patients, Procedures, and Radiation Dose

Fifty-four patients were enrolled in the study and randomly assigned to two groups, Group I and Group II, which consisted of 31 and 23 patients respectively. There were no adverse events reported. There were no significant differences between the two groups in sex, age, weight, height, scan time, or radiation dose (*P*> 0.05) as shown in Table 1. More patients received injections on the left side in Group II than Group I (*P*=0.001). (Table 1) 

**Table 1:** Study population characteristics and scan parameters in the two groups.

	Group I (n=31)	Group II (n=23)	*P* value
M/F**^*a*^**	21/10	16/7	0.887
Age (y) **^*b*^**	56.4±12.4	58.0±13.6	0.643
Weight (kg) **^*b*^**	65.3±10.1	69.6± 9.6	0.990
Height (m) **^*b*^**	1.66±0.063	1.66±0.065	0.122
Location of injection (left/right) **^*a*^**	15/16	20/3	0.001
Scan time (sec) **^*b*^**	3.04±0.14	3.02±0.19	0.727
CTDIvol (mGy) **^*b*^**	6.20±0.22	6.14±0.14	0.310
DLP (mGy×cm) **^*b*^**	294.5±20.7	293.3±16.0	0.224

Note: values represent mean ± standard deviation. CM = contrast medium

^a^ χ^2^ test ; ^b^ Student *t* test

### Quantitative assessment

In Group I, there were 11 occluded segments of arteries, including: C6 (n=1), MCA (n=2), V1 (n=1), V2 (n=1), V3 (n=3), and V4 (n=3). In Group II, there were seven occluded segments of arteries, including: MCA (n=1), V1 (n=2), V2 (n=2), V3 (n=1), and V4 (n=1). Table 2 lists the numbers of the different degrees of arterial stenosis and there was no significant difference between the two groups using the χ^2^ test (*P*=0.120). 

**Table 2:** Degrees of arterial stenosis in the two groups.

Arterial stenosis %	Group I	Group II
Mild (0-49)	1306	964
Moderate (50-69)	7	14
Severe (70-99)	9	4
Occluded (100)	11	7

**Figure 3:**
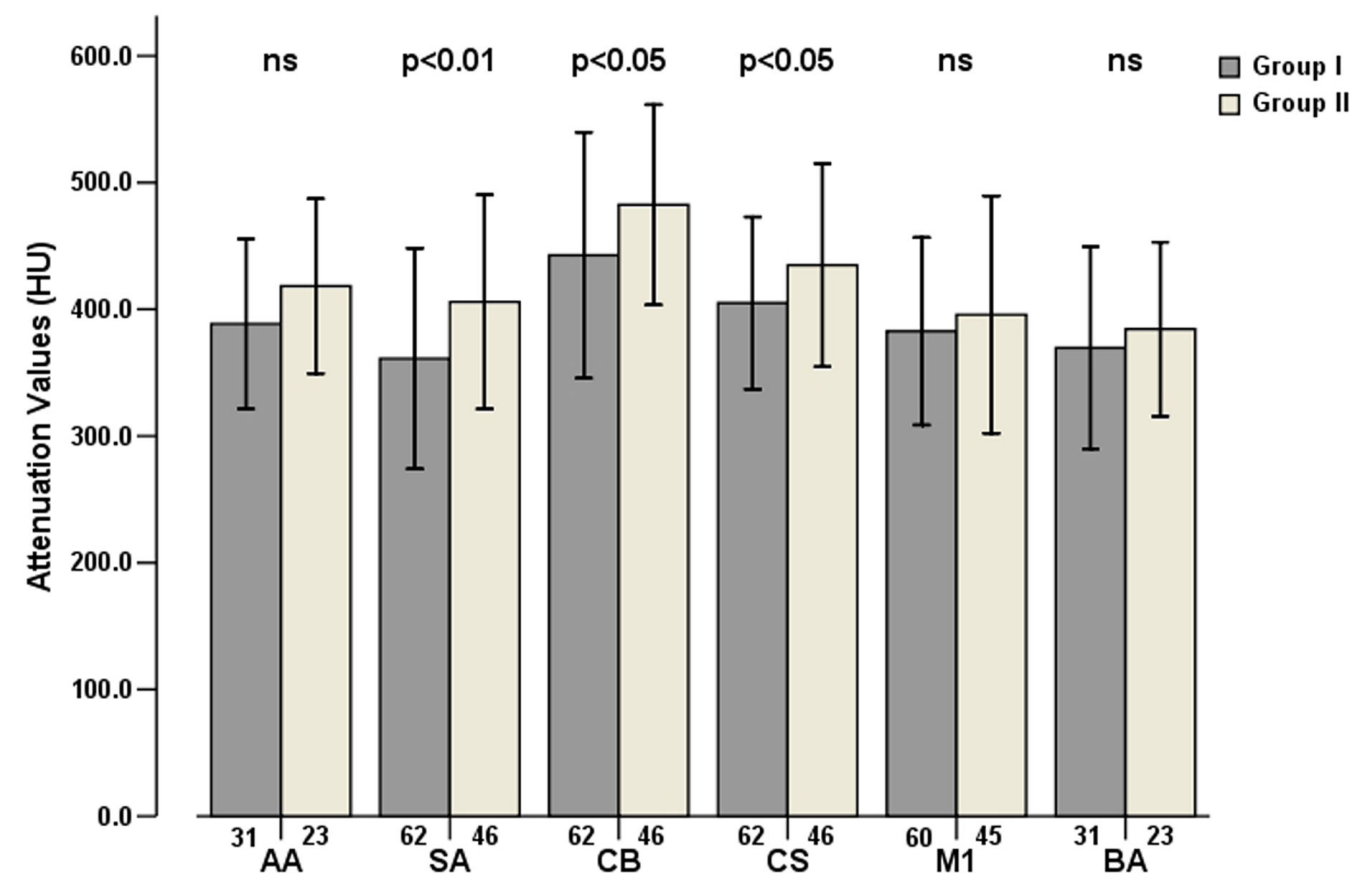
Mean arterial attenuation values and standard deviations (error bars) were measured at different locations in the two groups. The numbers under the bars represent the numbers of arterial segments of each location. Mean attenuation values of SA, CB and CS of Group II were significantly higher than those of Group I (*P*=0.009, 0.025, 0.039 respectively). AA=aortic arch, SA=subclavian artery, CB=bifurcation of common carotid artery, CS= carotid siphon, M=M1 segment of middle cerebral artery, BA=basilar artery.

**Figure 4:**
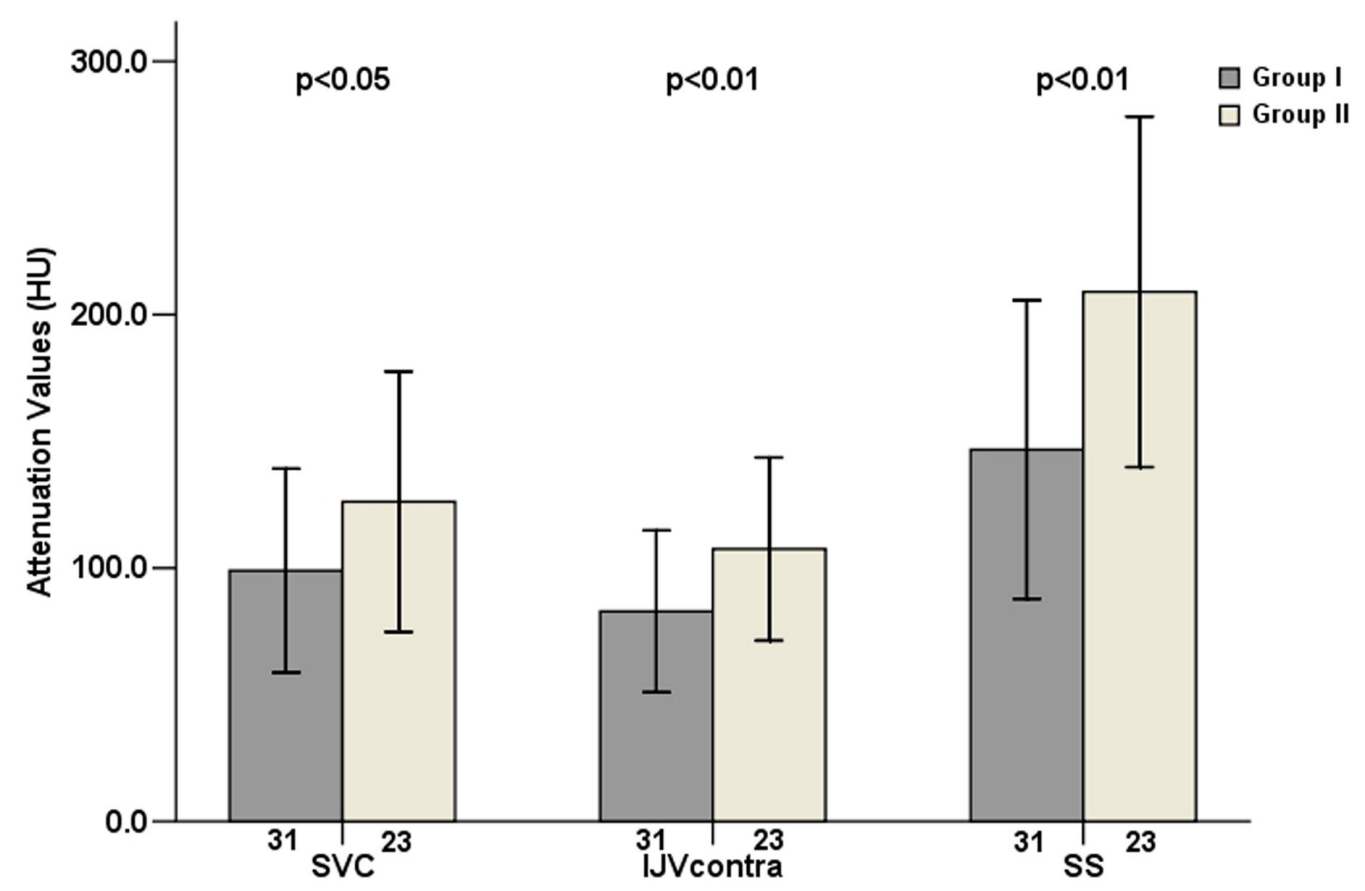
Mean venous attenuation values and standard deviations (error bars) were measured at different locations in the two groups. The numbers under the bars represent the numbers of venous segments of each location. Mean attenuations of SVC, IJV_contra_ and SS of Group II were significantly higher than those of Group I (*P*=0.034, 0.009, 0.001 respectively). SVC= superior vena cava, IJV_contra_=internal jugular vein on the contralateral side of contrast medium injection, SS=straight sinus.

**Figure 5:**
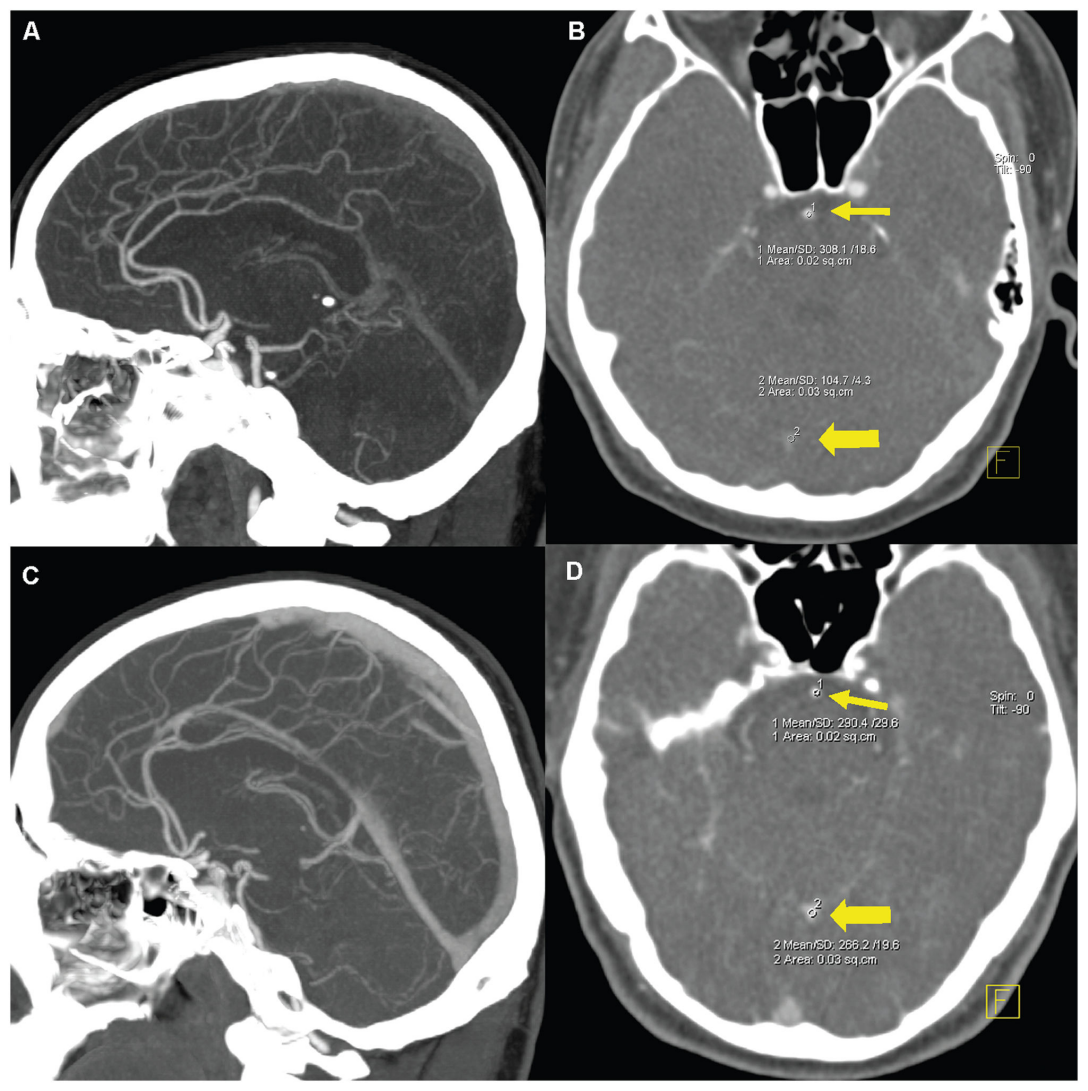
Examples of intracranial arterial and venous enhancement patterns in the two groups. Sagittal 25mm MIP (A) and transverse 1 mm MPR (B) of a patient from Group I show mild enhancement in intracranial veins. ROI-based measurement (B) reveals a vessel density of 104.7±4.3 HU in the SS (thick arrow) at the level of the BA (thin arrow) which had a vessel density of 308.1±18.6. Sagittal 25 mm MIP (C) and transverse 1 mm MPR (D) of a patient from Group II show marked venous enhancement of intracranial veins. The Vessel density of SS (D, thich arrow) is 266.2±19.6 HU at the level of the BA (thin arrow) which had a vessel density of 290.4±29.6. MIP= maximum intensity projection, MPR= multiplanar reformation, SS=straight sinus, BA=basilar artery.

Group II showed higher overall mean attenuation values (424.0±87.3 HU) obtained from arterial segments when compared with Group I (394.3±85.0HU) (*P*=0.000). However, Group II also had overall higher mean attenuation values of venous segments (146.6±68.9HU) compared with Group I (109.5±52.3HU) (*P*=0.000). Figures 3 and 4 show the segmental mean attenuation values of arteries and veins in both groups, including the levels of significances. Figure 5 illustrates examples of intracranial arterial and venous attenuation patterns in Group I and II.

### Qualitative Assessment

**Figure 6:**
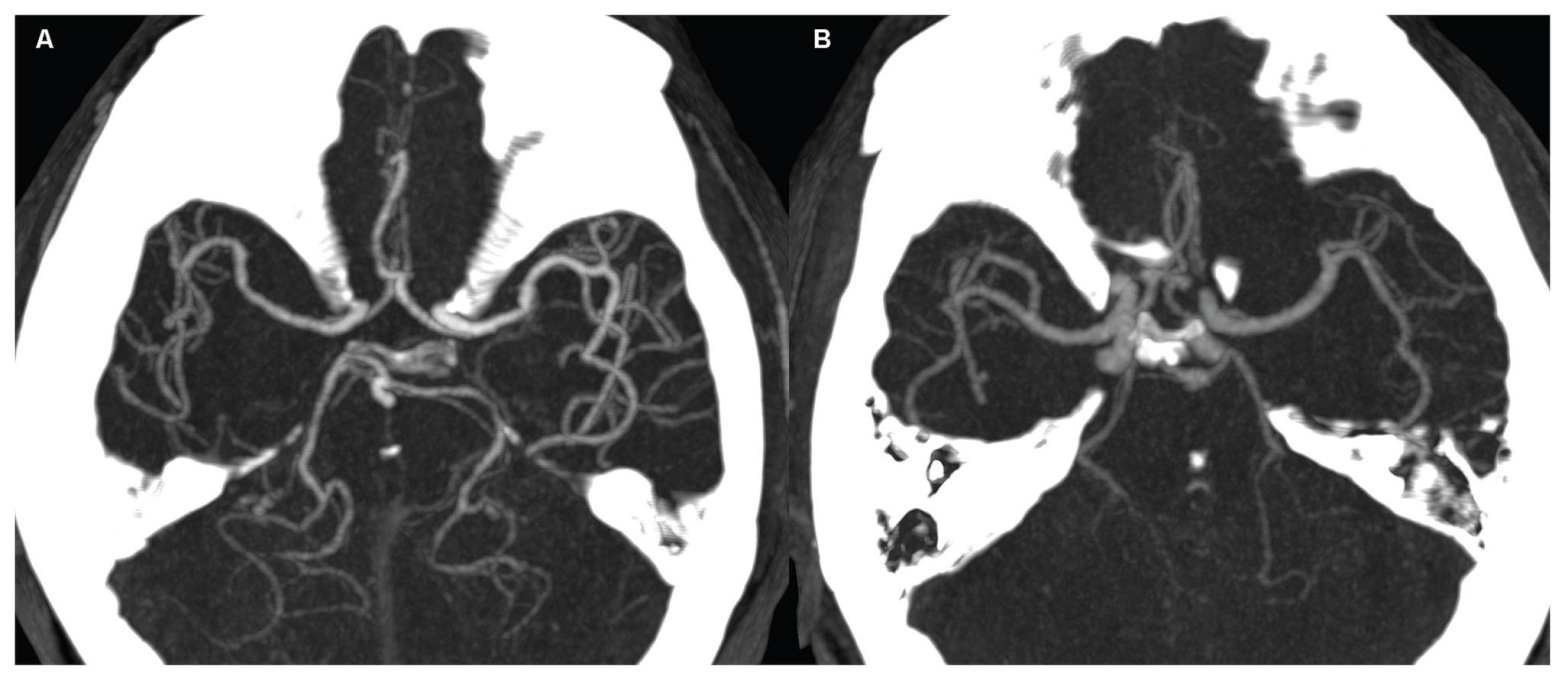
Examples of image quality of the Circle of Willis. (A) The 30 mm MIP image of a 66-year-old man weight 66 kg in Group II. The distal branches of the middle cerebral arteries are demonstrated clearly and it is considered as score 4. (B) The 30 mm MIP image of a 55-year-old man weight 85.5 kg in Group I. The branches of middle cerebral arteries are not clearly demonstrated and it is considered as score 3. MIP= maximum intensity projection.

Using the maximum intensity projections, no images of the Circle of Willis received S_willis_ 1 or S_willis_ 2 in either group. In Group I, the images of only three subjects were S_willis_ 3. The body weights of these patients were 85.5kg, 75.6kg and 81.0kg, respectively (Figure 6). All remaining images of the Circle of Willis in both groups were S_willis_ 4. The average scores of Group I and II were 3.90±0.30 and 4.00±0, respectively.

**Figure 7:**
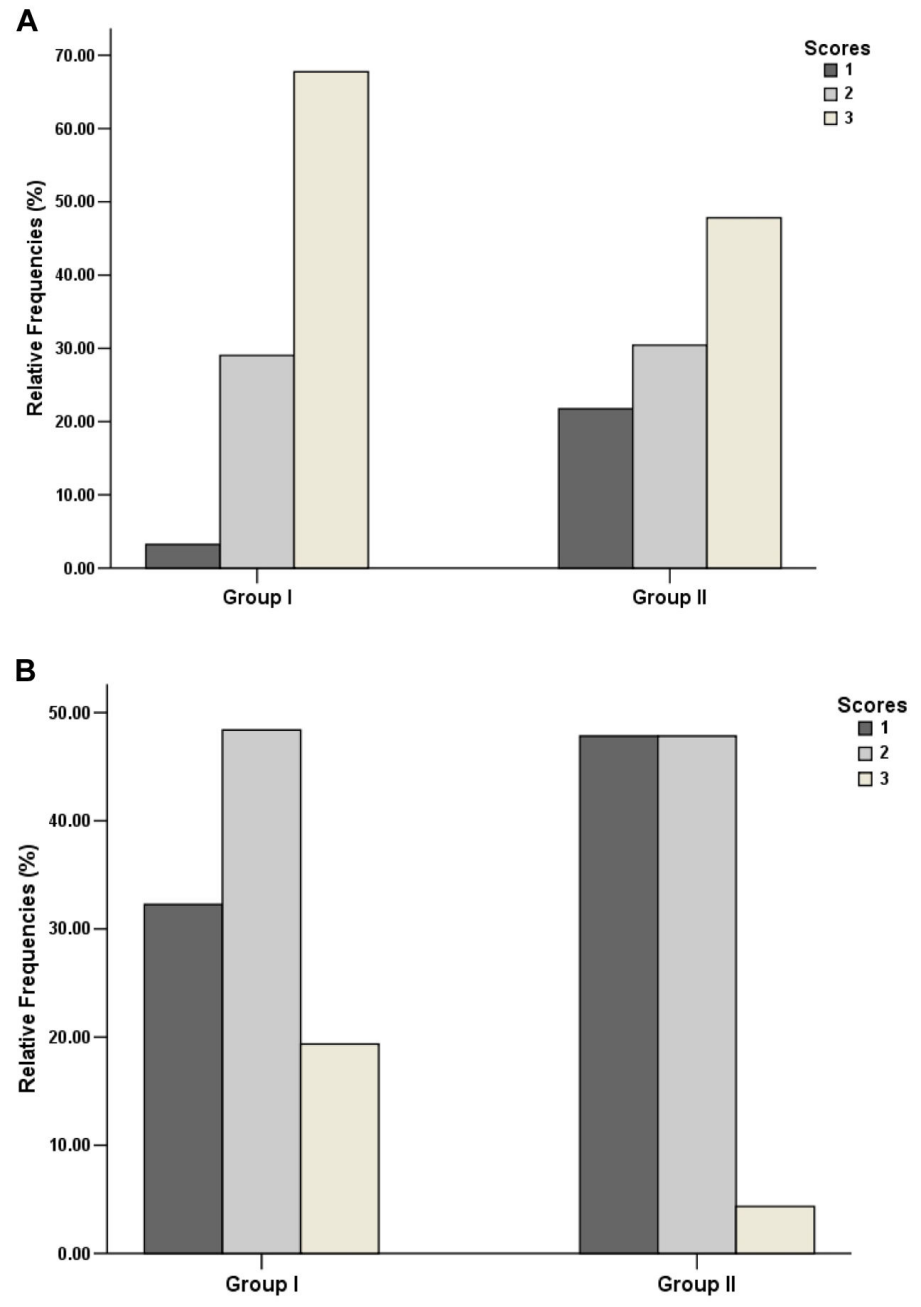
Qualitative evaluations of streak artifacts of residual CM on the ipsilateral side of injection in the two groups. (A) In Group I, the relative frequencies of score 1, 2 and 3 for the subclavian veins (SV_ipsi_) are 3% (1/31), 29% (9/31) and 68% (21/31), respectively. In Group II, the relative frequencies of score 1, 2 and 3 for the SV_ipsi_ are 22% (5/23), 30% (7/23) and 48% (11/23), respectively. (B) In Group I, the relative frequencies of score 1, 2 and 3 for the internal jugular veins (IJV_ipsi_) are 32% (10/31), 49% (15/31) and 19% (6/31), respectively. In Group II, the relative frequencies of score 1, 2 and 3 for the IJV_ipsi_ are 48% (11/23), 48% (11/23) and 4% (1/23), respectively. CM= contrast medium.

**Figure 8:**
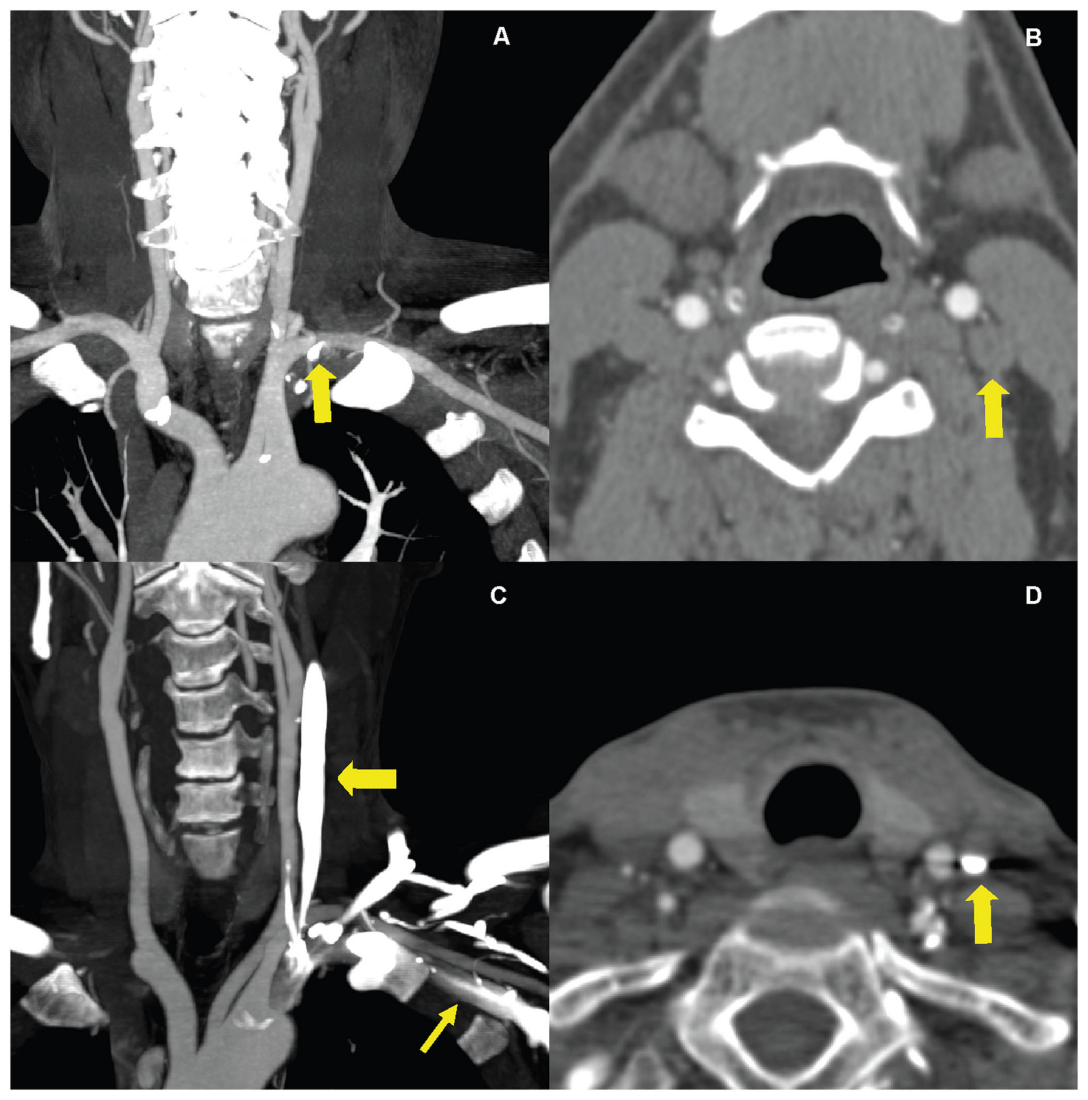
Examples of streak artifacts caused by residual CM in the veins on the ipsilateral side of injection. Coronal 25 mm MIP (A) of a patient from Group I shows a little residual CM in the left SV (arrow). His transversal 1 mm MPR (B) shows no residual CM in the left IJV (arrow). Coronal 25 mm MIP (C) and transversal 1 mm MPR (D) of a patient from Group II show large amount of CM in the left SV (thin arrow) and IJV (thick arrow) impairing evaluation of adjacent vessels. CM=contrast medium, MIP=maximum intensity projection, MPR=multiplanar reformation, SV= subclavian vein, IJV= internal jugular vein.

Qualitative scores of streak artifacts due to residual CM in the SV_ipsi_ and IJV_ipsi_ of the two groups are shown in Figure 7. The average scores of SV_ipsi_ were 2.65±0.55 and 2.26±0.81 for Group I and II, respectively. The average of IJV_ipsi_ were 1.87±0.72 and 1.48±0.51 for Group I and II, respectively. According to the χ^2^ test, relative frequencies of qualitative scores of SV_ipsi_ were not significantly different between the two groups (*P*=0.259). Fewer IJV_ipsi_ were S_vein_ 3 in Group II (4%, 1/23) than that of Group I (19%, 6/33) (*P*=0.021). Figure 8 illustrates examples of streak artifacts caused by residual CM in veins on the ipsilateral side of injection.

Excluding the occluded arterial segments, there were 1322 and 982 segments of arteries evaluated in Group I and Group II respectively for assessment of post-BR image quality. Excellent inter-observer agreements were found for assessment of bone remnants (κ= 0.836 for Group I; κ=0.847 for Group II) and vessel integrity after DE BR in both groups (κ= 0.931 for Group I; κ=0.949 for Group II). 

**Figure 9:**
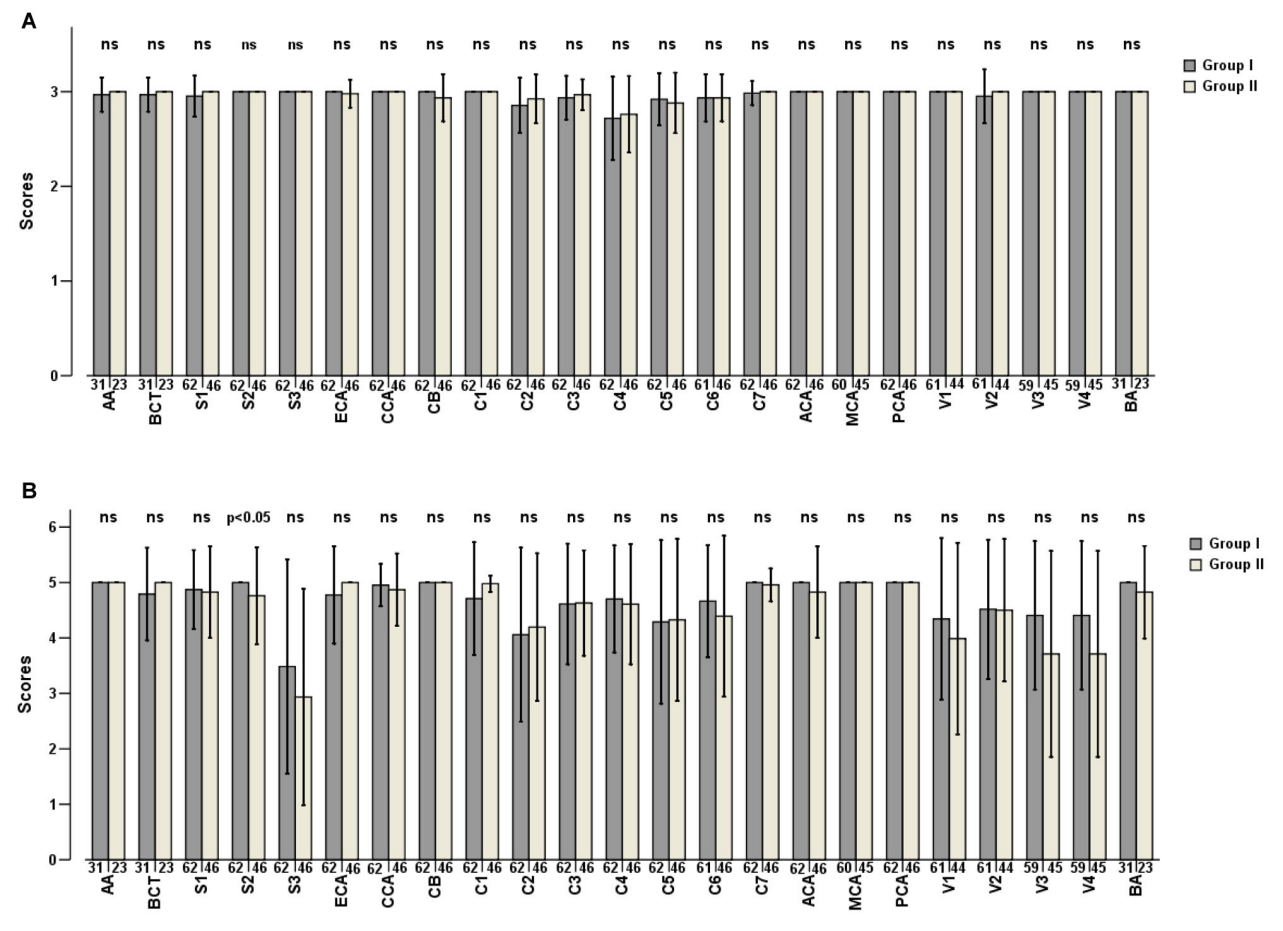
Mean scores and standard deviations (error bars) on evaluation of bone remnants (A) and vessel integrity (B) after dual-energy bone removal in the two groups. The numbers under the bars represent the numbers of arterial segments of each location. Group I has significantly higher mean scores of S2 segments for vessel integrity compared with that of Group II (*P*=0.019). AA=aortic arch, BCT=branchiocephalic trunk, S1-3=different segments of subclavian artery, ECA=external carotid artery and branches, CCA= common carotid artery, CB=bifurcation of CCA, C1-7=different segments of the internal carotid artery, ACA=anterior cerebral artery, MCA=middle cerebral artery, PCA=posterior cerebral artery, V1-4=different segments of the vertebral artery, BA=basilar artery.

Following the assessment of bone remnants after BR, the overall mean score of Group I was found to be 2.96±0.18 and 2.97±0.16 for Group II respectively, with no significant difference between the two groups (*P*=0.265). After assessment of vessel integrity following BR, the overall mean score of Group I (4.66±1.06) was significantly higher than that of Group II (4.54±1.22) (*P*=0.025). Figure 9 shows the scores of segmental bone remnants and vessel integrity in both groups averaged for both readers, including the corresponding levels of significances. 

## Discussion

Our results suggest that a protocol of low-volume CM for dual energy CTA of the head and neck is feasible with an acceletated-pitch 128-slice dual source CT system. The 40-mL protocol has lower venous attenuation and better image quality of post-BR vessel integrity as compared to the 50-mL protocol. In the literature, the smallest amount of CM used for a head and neck CTA used 30 mL of high-concentration iodinated contrast medium (370 mg I/mL) with a 128-slice CT system in their protocol. However in their study a single-energy protocol was used and a mean arterial enhancement of 243.3±39.4 HU was obtained (Hinkmann et al[14]). Although this mean arterial enhancement was suited for clinical diagnosis in Hinkmann’s research, it does not seem sufficient for the dual-energy bone removal. 

To our knowledge, the current study is the first to use less than 50 mL CM at dual energy CTA of supra-aortic arteries. 

Our data support the use of a reduction in the amount of CM from conventional methods currently used in head and neck CTA examinations. This reduction in CM would also decrease the costs involved in a head and neck CTA. Conventionally, 60-85 mL of high-concentration (≥350 mg I/mL) CM is needed to conduct a proper head and neck CTA in single energy CT and first-generation dual energy CT systems [23,24,19,25,26]. Our study proposes the use of 40-mL CM equivalent to the smallest available packaging of the medium (reducing the amount of CM by 33-53%) using the second-generation dual-energy CT system. 

We found the following protocols previously used in the literature: Thomas et al [23] used 70 mL of iodinated contrast medium (400 mg I/mL) at a 5 mL/s flow rate and a mean arterial enhancement of 410 HU (range, 215 HU~670 HU) was reached with a 64-slice dual-energy CT (Somatom Definition) system. Fujikawa et al [19] performed 60-mL and 80-mL CM of high iodine concentration (370 mg I/mL) at a 16 multidetector-row single-energy CT, which obtained an average arterial enhancement of 210.7 HU-383 HU and 310.3 HU-368.5 HU respectively and an average venous enhancement of 99.3 HU-132.1 HU in IJV and 304.3 HU-328.1 HU in SS. 

In addition several animal and clinical studies have demonstrated that increasing the injection rate produces a proportionally higher PME and faster tPME [27,28,29,30]. For this reason, we performed a faster rate of injection (6 mL/s) and higher arterial attenuation was reached while venous attenuation decreased due to the reduction of CM. The 40-mL group was found to have a lower mean venous attenuation and less residual CM in IJV on the ipsilateral side of injection than those of 50-mL group. Moreover, we attempted to decrease the amount of CM used for the test bolus to 5 mL, based on the faster rate of injection of 6 mL/s, which was significantly lower than those we found in the literature [14,31,15,32,33]. In our study, the average PME of a 5-mL test bolus was 176 HU at the bifurcation of the CCA. Therefore, the reduction in the amount of CM paired with the increase in rate of injection is shown to be sufficient in describing the time-concentration curves.

High vessel enhancement has been proposed to be an advantage, when differentiating bone from vessels filled with iodine [23]. Thomas C. et al [23] found a weak correlation between vessel enhancement and the performance of plaque and bone removal. Besides, there are several factors contributing to the mistaken deletion of vessel lumen following as a result of dual-energy bone removal. These include blooming artifacts caused by beam hardening of the calcified plaques [34], poor imaging results in very small vessel lumina due to large pixel size, and reduction of arterial enhancement in vessels with severe stenosis [23]. In our study however, there was no significant difference in arterial stenosis between the two groups.

Although the arterial enhancement of Group II was higher than that of Group I, mean scores of vessel integrity in Group I were higher than those of Group II, especially the S2 segments of subclavian arteries. Two further factors impairing post-bone-removal vessel integrity in subclavian arteries include first regions with high attenuation causing serious streaking artifacts by photon starvation [35] and second streak artifacts from CM ipsilateral to side of injection as described in our study. These factors can create errors during the post-processing of bone removal.

**Figure 10:**
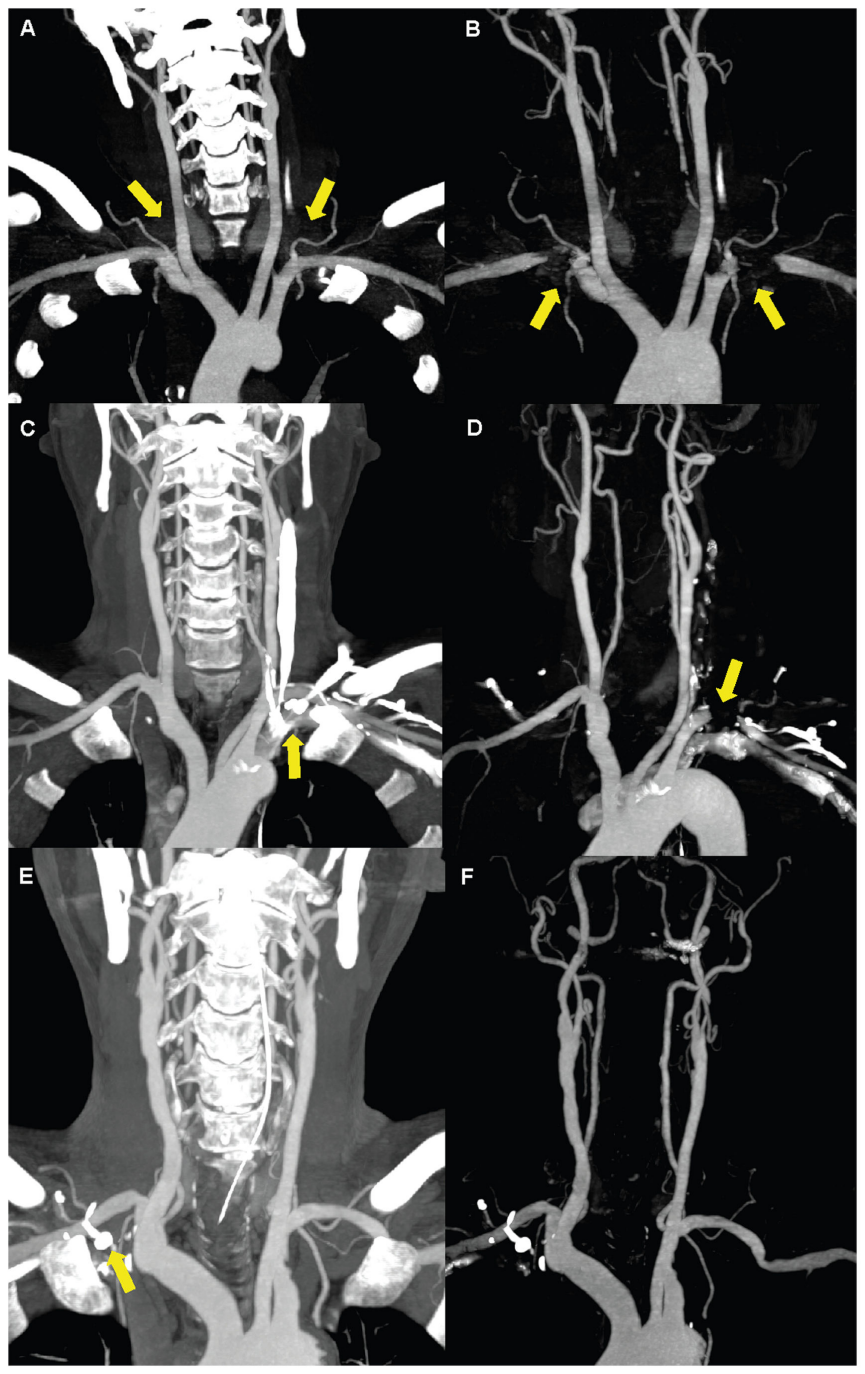
Examples of vessel integrity after dual-energy bone-removal postprocessing in the shoulder regions. The coronal 25 mm MIP before BR (A) of a 42-year-old man weight 85 kg in Group II shows significant streak artifacts in shoulders (arrow), and little residual CM in bilateral SV. His thick MIP image after BR (B) shows most of the bilateral S3 segments and left S2 segment (arrow) were deleted by mistake. The Coronal 25 mm MIP before BR (C) of a 48-year-old man weight 60 kg in Group II shows significant residual CM in the left SV (arrow), but the streak artifacts in shoulders were not obviously. His thick MIP image after BR (D) shows that the left S3 segment was deleted (arrow) by mistake. The coronal 25 mm MIP before BR (E) of a 53-year-old man weight 65 kg in Group I shows a little streak artifacts in shoulders and residual CM in the right SV (arrow). Arterial vessels in shoulders were complete in his thick MIP after BR (F). MIP= maximum intensity projection, BR=bone removal, CM= contrast medium, SV= subclavian vein, IJV= internal jugular vein. S1, S2 and S3=different segments of subclavian artery.

The proximal (S1) and middle (S2) segments of SA had the higher scores of vessel integrity compared with the distal (S3) segments in both groups (*P*=0.000 for both groups ). This illustrates that increasing the distance from the subclavian vein increases the attenuation of the vessel due to residual CM and lower attenuation of surrounding tissues. S3 segments had the lowest scores for vessel integrity which is most likely due to the lower attenuation of vessels, proximity to the residual CM in the SV, and higher attenuation of surrounding tissue. Although there was no significant difference of vessel intergrity between S1 and S2 in both groups (*P*=0.317 for Group I, *P*=0.433 for Group II), scores of the S2 segments were lower in Group II compared with Group I (*P*=0.019). It because that there were four S2 segments with lower scores (S_integrity_1 (n=2), S_integrity_3 (n=1), S_integrity_4 (n=1)) in Group II. And, all the S2 segments had score 5 in Group I. What is more, the S2 segments considered as S_integrity_1 and S_integrity_ 4 were all located on the ipsilateral side of CM injection. It was possible that residual CM had great influence to S2 segments in Group II because of more CM used. Figure 10 illustrates examples of vessel integrity after bone removal in the shoulder region.

We found two limitations in our study involving our patient population and the effect of cardiac output on enhancement. All patients enrolled in our study weighed less than 90 kg. A study based on a large Chinese population demonstrated that people 30 years and older had a mean body weight of 80.3 kg with a SD of 10.1 kg [36]. Most patients with suspected atherosclerotic diseases are expected to fall within that body weight group[37]. However, we are planning to evaluate the image quality of patients weighting more than 90kg with the current scan protocol in the future. Second, arterial enhancement would be expected to be inversely proportional to cardiac output[38,39]. In our study, the cardiac output factor was not explicitly quantified and we would need to do further research to take this into consideration.
